# Repeatability and reproducibility of apparent diffusion coefficient and fat fraction measurement of focal myeloma lesions on whole body magnetic resonance imaging

**DOI:** 10.1259/bjr.20200682

**Published:** 2021-02-22

**Authors:** Tara Barwick, Matthew Orton, Dow Mu Koh, Martin Kaiser, Andrea Rockall, Nina Tunariu, Matthew Blackledge, Christina Messiou

**Affiliations:** 1 Imperial College Healthcare NHS Trust and Imperial College, London, UK; 2 The Institute of Cancer Research, London, UK; 3 The Royal Marsden Hospital Foundation NHS Trust and The Institute of Cancer Research, London, UK; 4 The Royal Marsden Hospital Foundation NHS Trust, London, UK

## Abstract

**Objective:**

To assess intra- and inter-reader variability of apparent diffusion coefficient (ADC) and fat fraction (FF) measurement in focal myeloma bone lesions and the influence of lesion size.

**Methods:**

22 myeloma patients with focal active disease on whole body MRI were included. Two readers outlined a small (5–10 mm) and large lesion (>10 mm) in each subject on derived ADC and FF maps; one reader performed this twice. Intra- and inter-reader agreement for small and large lesion groups were calculated for derived statistics from each map using within-subject standard deviation, coefficient of variation, interclass correlation coefficient measures, and visualized with Bland–Altman plots.

**Results:**

For mean ADC, intra- and inter-reader repeatability demonstrated equivalently low coefficient of variation (3.0–3.6%) and excellent interclass correlation coefficient (0.975–0.982) for both small and large lesions. For mean FF, intra- and inter-reader repeatability was significantly poorer for small lesions compared to large lesions (intra-reader within-subject standard variation estimate is 2.7 times higher for small lesions than large lesions (*p* = 0.0071), and for inter-reader variations is 3.8 times higher (*p* = 0.0070)).

**Conclusion:**

There is excellent intra- and inter-reader agreement for mean ADC estimates, even for lesions as small as 5 mm. For FF measurements, there is a significant increase in coefficient of variation for smaller lesions, suggesting lesions >10 mm should be selected for lesion FF measurement.

**Advances in knowledge:**

ADC measurements of focal myeloma have excellent intra- and inter-reader agreement. FF measurements are more susceptible to lesion size as intra- and inter-reader agreement is significantly impaired in lesions less than 10 mm.

## Introduction

Over the past decade, whole body MRI (WB-MRI) has emerged as the most sensitive technique for detecting focal active myeloma lesions within bone marrow. As a consequence, the International Myeloma Working Group (IMWG) consensus guidelines recommend WB-MRI for all patients with a suspected diagnosis of asymptomatic myeloma and apparently solitary plasmacytoma where sensitivity and detection of focal lesions influences management.^1^ In the UK, The National Institute for Health and Care Excellence (NICE) has recommended WB-MRI as first line imaging for assessment of all patients with a suspected diagnosis of myeloma or relapse/progression.^2^ The British Society of Haematology also recommends WB-MRI for monitoring response of non/oligo secretory myeloma and patients with extramedullary disease.^3^

Incorporating diffusion-weighted imaging (DWI) into WB-MRI protocols further increases sensitivity but also enables qualitative and quantitative assessments of response using apparent diffusion coefficient (ADC) measurements.^4–7^ Gradient-echo based Dixon MRI has also become embedded in contemporary WB-MRI protocols.^8,9^ Dixon MRI enables quick anatomical display, and quantitative measures of the proportion of fat and water in bone marrow, measured as percentage fat fraction (FF), also give insight into disease status.^4,10^ Response to treatment leads to an increase in FF of involved bone marrow^4,11^ and early changes in lesional FF has also been shown to predict response to chemotherapy in newly diagnosed myeloma patients.^12^

Studies assessing reproducibility of ADC measurement of other tumour sites have shown that lesion size has a significant impact.^13,14^ However, to date no study has assessed whether there is a minimum lesion size for which accurate and repeatable/reproducible ADC and FF measurements in bone marrow can be measured by observers. This is important as the IMWG criteria for the diagnosis of myeloma include focal lesions on MRI ≥ 5 mm as a myeloma defining event.^1^ ADC measurements are also incorporated into the recent myeloma response assessment and diagnosis system (MY-RADS) guidelines for WB-MRI in myeloma.^9^

The primary aim of this study is to establish the intra- and inter-reader agreement of ADC and FF measurement in focal active myeloma bone marrow deposits, and whether a lesion size cut-off acceptable for quantitative measurement can be defined.

## Methods

Institutional review board approval was obtained, and all patients provided written informed consent.

### Patient cohort

22 sequential patients with a new established diagnosis of myeloma with focal active disease on WB-MRI as per International Myeloma Working Group criteria^1^ were prospectively included. Based on existing literature^8^ and a pilot evaluation performed as part of the study reported here, it was determined that evaluating a size threshold of 10 mm was suitable for this study.

The purpose of the pilot evaluation was to provide preliminary estimates of interobserver agreement in small and large lesions (which are not currently known), to guide the selection of a suitable size threshold, and thereby to power the sample size of the main study. In this pilot study, 34 lesions (size range 2–55 mm) from 4 subjects (Table 1) not included in the main study were outlined by 2 observers, yielding estimates of mean ADC and mean FF for each lesion. Interobserver agreement (standard deviation of differences in ADC and FF) was assessed for small lesions and for large lesions, as determined by a size threshold. This threshold was varied between 2 and 55 mm, and it was found that for thresholds between 8 and 20 mm the interobserver standard deviation was around two times higher for lesions below the size threshold compared with lesions above the threshold. As this estimate of the standard deviation ratio is determined by performing multiple comparisons, a meaningful *p*-value cannot be easily computed, hence the use of these pilot data to power a larger study in an independent patient cohort. On the basis of this pilot study, it was determined that a size threshold of 10 mm was likely to yield a measurable and meaningful difference in observer agreement, and based on existing literature,^8^ this size threshold is also clinically relevant. In the main study, differences of observer agreement between small and large lesions are assessed using Levene’s test for equality of variances,^15^ and for a power of 0.8 and a significance level of 0.05 a sample size of 22 patients (yielding 22 small and 22 large lesions) is required to detect a repeat measures standard deviation ratio of 2 between the small and large lesions. This sample size calculation was performed using a Monte Carlo simulation written in MATLAB (v. 9.6.0 (R2019a). Natick, MA: The MathWorks Inc.) according to the methods,^16^ and a sample size of 100,000 was used to ensure convergence of the simulation.

**Table 1.** Number, location and size category of lesions selected from four patients in the pilot evaluation

Site of lesion	Number of lesions	Small (<10 mm) Range 2–9 mm	Large (≥10 mm) Range 10–55 mm
Skull	0	0	0
C spine	0	0	0
T spine	3	1	2
L spine	4	3	1
Sternum	2	1	1
Clavicle	1	0	1
Ribs	7	2	5
Scapula	3	2	1
Humerus	1	0	1
Pelvis	8	4	4
Femur	5	5	0
Total	34	18	16

### Image acquisition

WB-MRI studies were performed using an Avanto 1.5 T system (Siemens, Erlangen, Germany). All subjects were scanned supine with arms by their sides. Coil elements were positioned from skull vertex to knees. See Table 2 for sequence parameters. No intravenous gadolinium contrast was used. ADC maps were generated using a mono-exponential fit using the scanners proprietary software. Fat fraction (FF) maps were produced from the water only (WO) and fat only (FO) Dixon sequences: FF = FO/(FO +WO).^4^

**Table 2.** Image acquisition parameters

Sequence	T1	T2	DWI	Dixon
Orientation	Sagittal	Sagittal	Axial	Axial
Repetition/echo time (ms)	590/11	2690/93	14800/66	7/2.38
FOV (mm)	400	400	430	470
Slice thickness (mm)	4	4	5	5
Flip angle (^0^)				30
b values (s mm^−2^)			50 600 900	

DWI, diffusion-weighted imaging; FOV, field of view.

### Quantitative analysis

**Figure 1.**
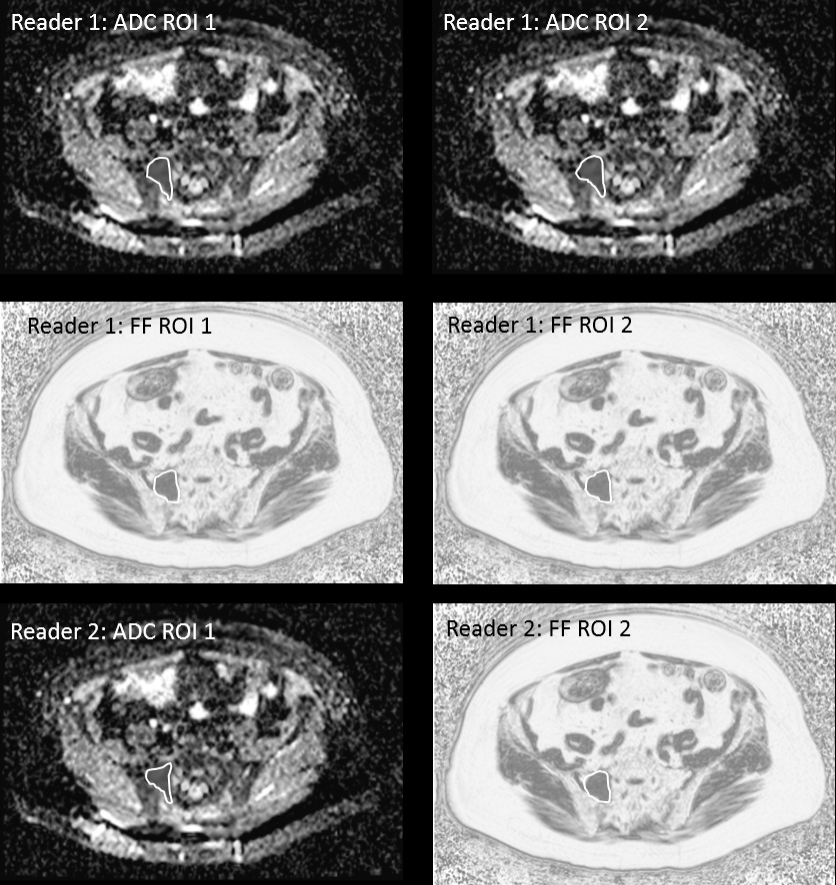
Regions of interest drawn on a lesion >10 mm in the right side-of the sacrum. Reader one has drawn the ROI twice on the ADC map on two separate occasions and twice on the FF map on two separate occasions. Reader two has drawn the ROI on the ADC and FF maps. This process is repeated for a small lesion (<10 mm) within the same patient. ADC, apparent diffusion coefficient; FF, fat fraction; ROI, region of interest.

Two radiologists, each with more than 15 years’ experience of MRI reporting, reviewed the images. All measurements were performed on a PACS workstation (Sectra). The presence of focal active lesions in any part of the imaged volume was confirmed by consensus. A focal active site of disease was confirmed as a focal marrow lesion which was hyperintense to background marrow and muscle on b900 s mm^−2^ images, with an intermediate ADC and corresponding focal abnormality on Dixon imaging.^9^ For each patient, a small lesion (5–10 mm) and a large lesion (≥10 mm) were selected avoiding areas degraded by artefact. Both lesions were outlined separately on ADC and FF maps on the equivalent axial slice with the maximum lesion diameter using the ROI tool to manually contour around the periphery of the lesion.^12^ This was performed twice by one reader (blinded to the first), and once by a second reader (blinded to the first) (Figure 1), resulting in six regions of interest (ROIs) per subject, per image contrast. Estimates of the mean (ADC-Mean and FF-Mean) and standard deviation (ADC-SD and FF-SD) of the pixel values over each ROI were recorded, in addition to the ROI area for each contrast (ADC-Area and FF-Area).

### Statistics

Intra- and inter-reader differences were calculated for all estimates, and Bland–Altman plots^17^ were used to visually assess the distribution of differences (spread of points along y-axis), and to compare the distribution of estimates obtained for small and large lesions (spread and separation of points along x-axis). Within-subject standard deviations (*s_w_*) were calculated using

$$s_{w}=\sqrt{\frac{1}{2N}\sum_{n=1}^{N}{\left(d_{n}-\bar{d}\right)}^{2}}$$

where $d_{n}$ is the difference between two estimates for subject n, and $\bar{d}=N^{-1}\sum_{n=1}^{N}d_{n}$ is the mean difference over all subjects (which is equivalent to the within-subject standard deviations from an ANOVA model.^18^ Differences were computed using $d_{n}=x_{1,n}-x_{2,n}$, where $x_{1,n}$ and $x_{2,n}$ are two estimates of the same lesion for subject n; either the first and second estimates from Reader 1, or the first estimates from readers 1 and 2. Levene’s test^15^ was used to determine if the within-subject variances (${s_{w}}^{2}$) obtained for small and large lesions were significantly different. This test takes the ADC or FF differences from the small and large lesion groups as input, and was chosen to be robust to any departures in normality.^19^

The group mean values of all statistics for small and large lesions were also computed, using the mean of the three estimates for each lesion (two from one reader, one from the second reader), and the presence of significant differences between the two size groups was evaluated using paired *t*-tests.

Within-subject standard deviations were converted to coefficients of variation (CoV) by dividing by the corresponding group mean and multiplying by 100%.

Intraclass correlation coefficients (ICCs) of ADC and FF estimates for small and large lesions were calculated using the ICC^1,1^ formula in reference^20^ (including the calculation of 95% confidence interval),^20^ and ICC values less than 0.5 suggest poor agreement, 0.5–0.75 moderate, 0.75–0.9 good and greater than 0.9 excellent agreement.^21^ The ICC is an index that informs on the ratio between measurement variability and inter patient variability, such that large values of ICC indicate the measurement variability is much lower than the interpatient variability. It is therefore particularly useful for determining if a given measure (*e.g.* lesion mean ADC) can be used to differentiate between different patients in a given cohort.

All statistical calculations were performed using MATLAB (v. 9.6.0 (R2019a). Natick, MA: The MathWorks Inc.).

## Results

**Figure 2.**
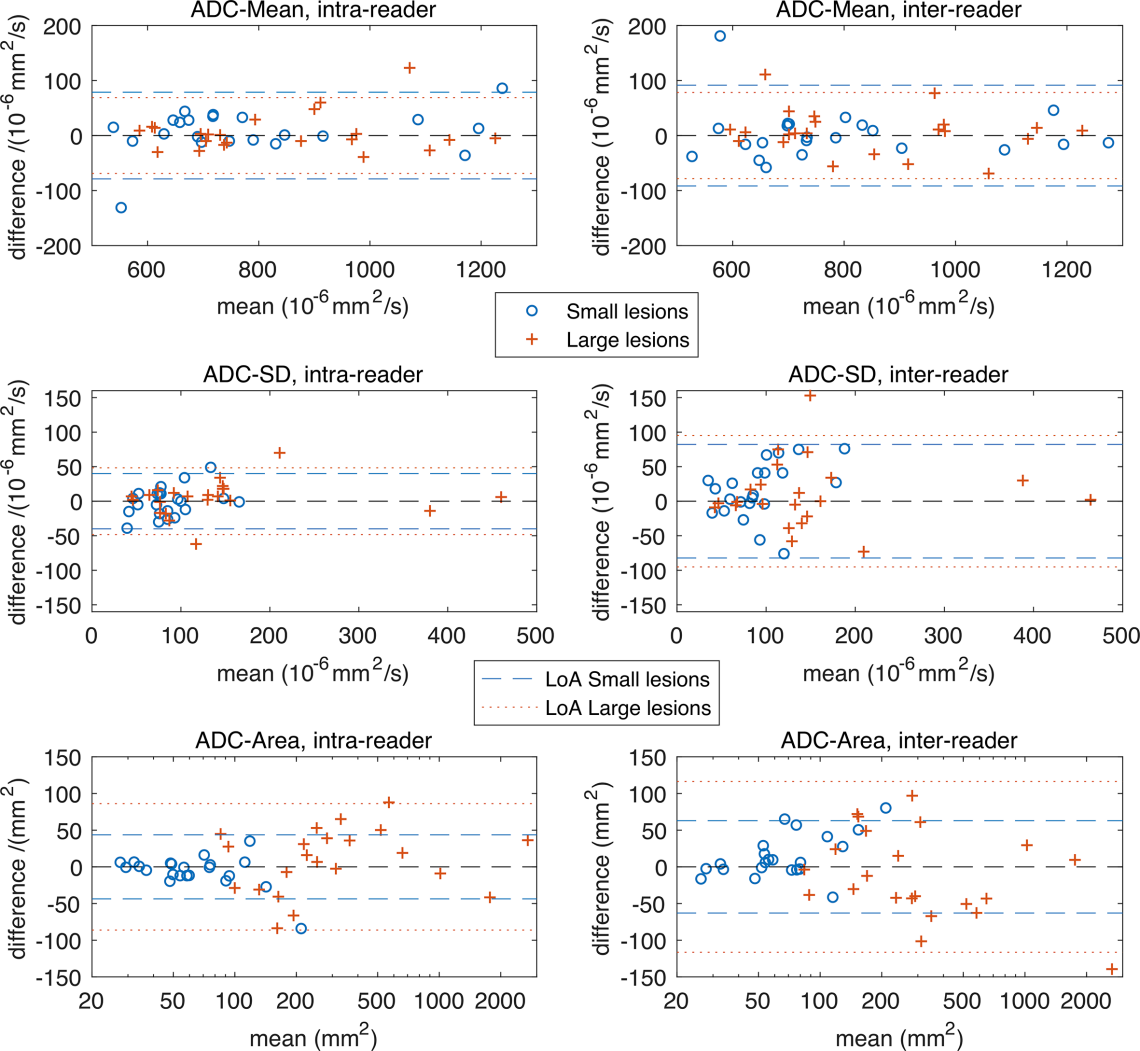
Bland–Altman plots for estimates derived from ADC maps. Plots in the top row are for intra- and inter-reader differences in ADC-Mean, the middle row are for ADC-SD and the bottom row for ADC-Area. LoA are shown as dashed and dotted lines, where LoA =±1.96 $\sqrt{2}$ s_w_ and gives a 95% confidence interval on the difference between two measurements that would be expected when there is no change in the underlying parameter. Observed differences outside of this interval would indicate a statistically significant change in the underlying parameter. The plots show similar repeat measures variation in ADC-Mean for small and large lesions, and for intra- and inter-reader differences. A similar pattern is seen for ADC-SD, although there are a few large lesions with higher ADC-SD values, suggesting a potential link between lesion size and heterogeneity. As expected, the ADC-Area of the large lesions is higher on average than small lesions (the overlap on this figure is because the small v.s. large classification is based on diameter, not area), and the repeat measures variation for intra- and inter-reader differences is visibly larger for large lesions (*p* < 0.01, see Table 4). Table 4 also expresses these differences in ADC-Area as CoV/% values, and on this relative scale intra- and inter reader differences are smaller for larger lesions than small lesions. ADC, apparent diffusion coefficient; CoV, coefficient of variation; LoA, limits of agreement.

**Figure 3.**
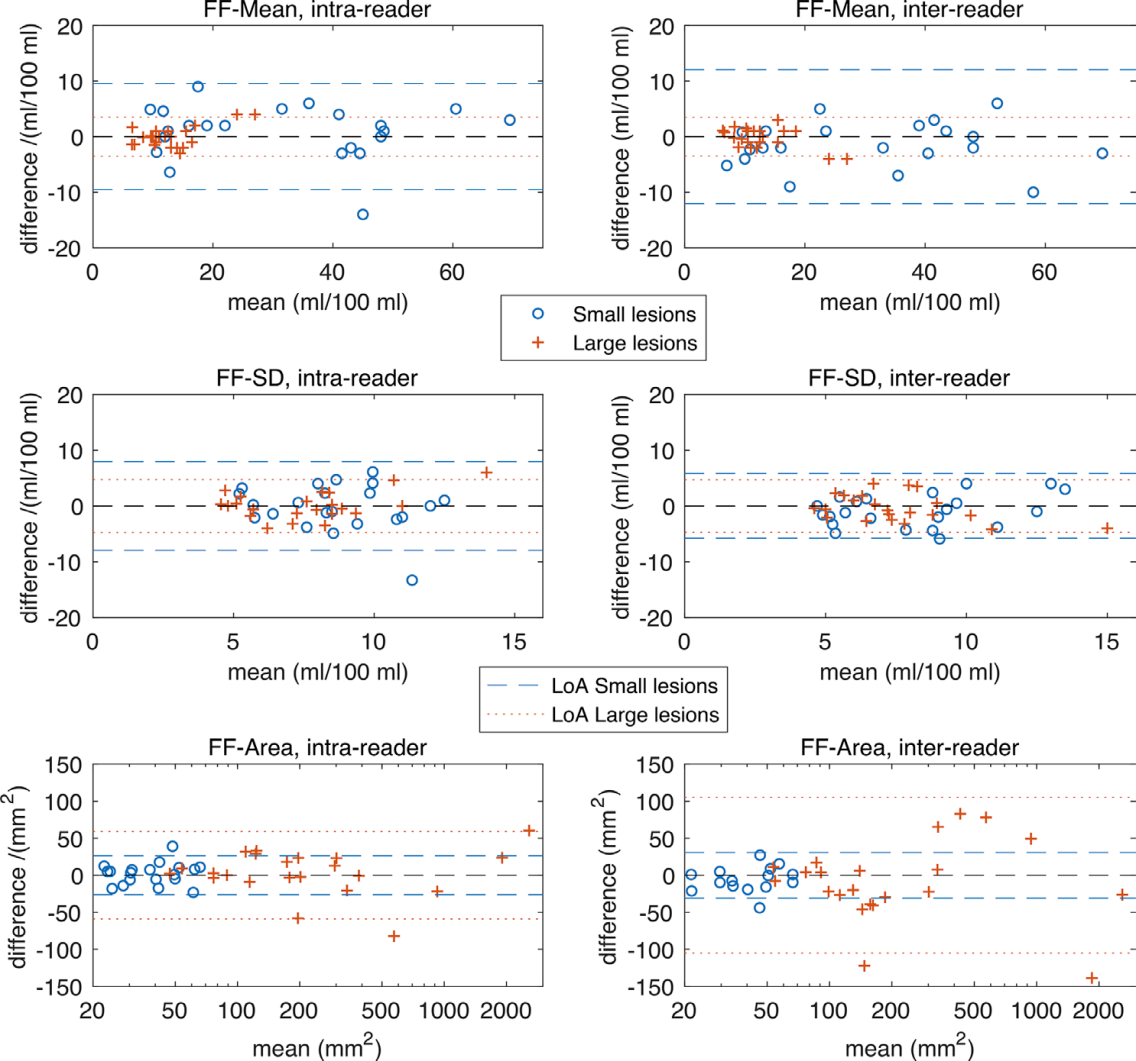
Bland–Altman plots for estimates derived from FF maps. Plots in the top row are for intra- and inter-reader differences in FF-Mean, the middle row are for FF-SD and the bottom row for FF-Area. LoA are shown and are as described in Figure 2. The FF-Mean values for small lesions are higher than for large lesions, and this may be due to the difficulty of boundary localization in small lesions on the FF images. The intra- and inter-reader differences in FF-Mean are significantly larger for the small lesions compared with large lesions, see Table 4 for statistical details. For the FF-SD, there are no clear visible differences in these plots between small and large lesions, or between intra- and inter-reader differences, although the intra-reader differences for small lesions are around 70% larger than those for large lesions (*p* = 0.0374). As with ADC-Area, the FF-Area shows expected patterns, and similarly Table 4 indicates that the intra- and inter-reader *relative* differences are smaller for larger lesions. CoV, coefficient of variation; FF, fat fraction; LoA, limits of agreement.

22 sequential patients with a new established diagnosis of myeloma with focal active disease on WB-MRI were recruited (15 male,7 female, mean age 58.7 years, age range 31–72). The number, location and size category of marrow lesions in these patients are shown in Table 3. Figures 2 and 3 show Bland–Altman plots for intra- and inter-reader differences for all ADC and FF estimates, and Table 4 gives estimates and statistics relating to the within-subject standard deviations (*s_w_*), group means and ICCs.

**Table 3.** Number, location and size category of lesions selected from 22 patients

Site of lesion	Number of lesions	Small (<10 mm) Range 3–9 mm	Large (≥10 mm) Range 10–53 mm
Skull	1	1	0
C spine	0	0	0
T spine	6	3	3
L spine	5	3	2
Sternum	1	1	0
Clavicle	2	1	1
Ribs	4	2	2
Scapula	4	1	3
Humerus	5	2	3
Pelvis	12	4	8
Femur	4	4	0
Total	44	22	22

**Table 4.** Within-subject standard deviations (*s_w_*), also given as CoV in parenthesis, group means, mean difference between measurements and ICC of ADC and FF estimates for small and large lesions

		s_W_ (CoV/%)					Group mean			Mean difference between measurements				ICC			
		Small		Large		*p* value	Small	Large	*p* value	Small		Large		Small		Large	
ADC-Mean	Intra	28.4	(3.62)	24.8	(2.98)	0.712	791	839	0.391	−6.91	(−23.9, 10.0)	−5.18	(−20.0, 9.68)	0.981	(0.955, 0.992)	0.982	(0.959, 0.993)
(10^−6^ mm^2^ s^−1^)	Inter	23.5	(3.04)	28.5	(3.42)	0.735				−2.87	(−22.8,17.1)	−6.45	(−23.3, 10.4)	0.975	(0.941, 0.989)	0.977	(0.946, 0.990)
ADC-SD	Intra	14.4	(15.9)	17.4	(11.8)	0.984	90.0	143	0.0147	1.32	(−7.39, 10.0)	−4.55	(−14.9, 5.82)	0.814	(0.608, 0.918)	0.969	(0.928, 0.987)
(10^−6^ mm^2^ s^−1^)	Inter	26.9	(30.1)	30.6	(20.7)	0.888				−15.0	(−31.8, 1.71)	−9.95	(−30.3, 10.4)	0.553	(0.188, 0.785)	0.884	(0.745, 0.950)
ADC-Area	Intra	15.8	(20.9)	31.1	(6.52)	0.00107	75.3	479	0.00579	6.00	(−3.19, 15.2)	−9.18	(−27.6, 9.21)	0.872	(0.721, 0.945)	0.997	(0.994, 0.999)
(mm^2^)	Inter	17.7	(22.6)	37.1	(7.72)	0.00387				−14.2	(−26.5, 1.87)	11.3	(−13.6, 36.2)	0.759	(0.510, 0.892)	0.995	(0.989, 0.998)
FF-Mean	Intra	3.44	(11.4)	1.27	(9.94)	0.00713	31.2	12.8	0.000246	−0.923	(−2.97, 1.12)	0.0318	(−0.737, 0.801)	0.963	(0.914, 0.984)	0.937	(0.857, 0.973)
ml/100 ml	Inter	3.89	(12.2)	1.04	(8.10)	0.00705				2.396	(−0.0256, 4.82)	0.241	(−0.510, 0.992)	0.940	(0.863, 0.975)	0.939	(0.863, 0.974)
FF-SD	Intra	2.87	(34.8)	1.71	(22.7)	0.0903	8.32	7.53	0.164	0.209	(−1.52, 1.94)	−0.155	(−1.19, 0.879)	0.037	(0.0001, 0.441)	0.568	(0.210, 0.794)
ml/100 ml	Inter	2.40	(29.3)	1.40	(19.1)	0.0374				0.886	(−0.318, 2.09)	0.341	(−0.679, 1.36)	0.538	(0.167, 0.777)	0.565	(0.205, 0.792)
FF-Area	Intra	9.48	(26.8)	21.3	(5.18)	0.0303	35.1	409	0.0113	−1.67	(−7.35, 4.02)	−3.12	(−15.9, 9.69)	0.683	(0.383, 0.854)	0.999	(0.997, 0.999)
(mm^2^)	Inter	8.14	(23.0)	35.1	(8.62)	0.000944				5.85	(−0.369, 12.1)	9.81	(−12.8, 32.4)	0.648	(0.327, 0.836)	0.996	(0.991, 0.998)

ADC, Apparent diffusion coefficient; CoV, coefficients of variation; FF, fat fraction; ICC, intraclass correlation coefficient.

Comparisons of *s_w_* between small and large lesions are from Levene’s test, and *t*-tests are used to compare small and large lesion group means. Intra = intra-reader-reader; Inter = inter-reader-reader. Pairs of numbers in parentheses are 95% confidence intervals.

For ADC-Mean, the Bland–Altman plots show that the intra- and inter-reader differences are similar when compared between large and small lesions, and this is confirmed by the statistics in Table 4, where the *p*-values from Levene’s test fail to reach significance in both cases. The CoV values are low (3.0–3.6%) with excellent intraclass correlation coefficients (ICC = 0.975–0.982). The corresponding visual comparisons of the group mean values and estimates of *s_w_* are also similar, and this is confirmed by the non-significant *t*-test for ADC-Mean differences.

The intra- and inter-reader differences for ADC-SD for large and small lesions are similar, with Levene’s test returning non-significant *p*-values, whereas the group mean comparisons show that ADC-SD is higher by 60% in larger lesions (*p* = 0.015). Inter-reader variability is higher than intra-reader variability for both size groups – around 86% higher for small lesions (inter-reader *s_w_* 26.9 *vs* intra-reader *s_w_* 14.4) and 75% higher for small lesions (inter-reader *s_w_* 30.6 *vs* intra-reader *s_w_* 17.4).

As expected, the ADC-area group mean for larger lesions is significantly higher than for smaller lesions, although there is some overlap on the Bland–Altman plots. This is because the area of any elongated ROIs in the large group (according to longest diameter) can be smaller than ROIs in the small group that are more circular. For intra- and inter-reader differences, *s_w_* for large lesions is around double that for small lesions (intra reader *s_w_* 31.1 large lesions *vs* 15.8 small lesions; inter reader *s_w_* large lesions 37.1 *vs* 17.7 small lesions).

For the FF measurements in general, the CoV values are higher than the corresponding ADC measurements. For example, for FF-Mean CoV values are in the range 8.1–12%, whereas ADC-Mean CoV values are in the range 3.0–3.6% (Table 4).

For FF-mean, the Bland–Altman plots show greater variation for small lesions than larger lesions on both intra- and inter-reader analysis (Figure 2). The corresponding intra-reader *s_w_* for FF-Mean estimates is 2.7 times higher for small lesions than large lesions (3.44 small *vs* 1.27 large; *p* = 0.0071), and for inter-reader variations is 3.8 times higher (3.89 small *vs* 1.04 large; *p* = 0.0070). Group mean statistics also show significant differences between FF-Mean estimates for small and large lesions, with values of around 32 ml/100 ml for small lesions, and around 13 ml/100 ml for large lesions. It is interesting to note that for small lesions, the group mean FF-Area is around half that of the corresponding ADC-Area group mean (group mean FF-Area 35.1 mm^2^ *vs* group mean ADC-Area 75.3 mm^2^) but only 15% lower for large lesions (group mean FF-Area 409 mm^2^ *vs* group mean ADC-Area 479 mm^2^).

Estimates of FF-SD variations are a little higher for small lesions than large lesions, although this only reaches significance for inter-reader variations (*p* = 0.037). Group mean values of FF-SD for large and small lesions are not significantly different. Inter-reader variations in FF-Area are around four times higher for large lesions than small lesions

The ICC values are excellent for FF-mean (0.940–0.963) in both large and small lesions. However, the ICC values for FF-SD are poor to moderate, and for FF-Area are moderate for small lesions but excellent for large lesions.

## Discussion

WB-MRI with DWI has become established as the most sensitive technique for bone marrow imaging and measurement of changes in lesion ADC and FF are useful for response assessment. Currently in clinical practice, this is usually assessed visually, however, there is a move towards quantitative assessment. Repeatability and reproducibility have been defined by the Imaging biomarker roadmap for cancer studies as a crucial element of technical validation. This should be performed initially in single/small number of centres but in multiple centres at later stages of biomarker development.^22^ Assessments of precision are crucial to determine if differences measured in patients fall outside limits of repeatability and reproducibility.

The measurement of ADC in normal bone marrow and diffuse disease has good to excellent reproducibility. However, observer agreement of ADC and FF measurement in focal lesions and the influence of lesion size has not been previously explored. This is important as the IMWG criteria stipulate 5 mm as the threshold for defining unequivocal focal active disease.^1^

Repeatability of ADC measurement of bone marrow in healthy volunteers has been assessed within previous small studies, performed by a single observer on two sets of images from the same subjects scanned a short interval apart. A repeatability study of mean ADC derived from bone marrow in nine healthy volunteers reported a coefficient of variation of 14.8%.^23^ Repeatability of ADC and FF of bone marrow in 10 healthy volunteers, scanned up to 4 weeks apart, showed that FF had excellent repeatability (ICC of 0.98) compared with moderate repeatability of ADC (ICC of 0.47).^24^ Whole marrow ADC segmentations have produced impressive results of 3.8% coefficient of variation in normal volunteers and 2.8% in myeloma patients.^6^ However, the inter-reader agreement of ADC and FF measurements in focal marrow lesions has not been previously explored. On a per lesion basis, intra- and inter-reader variability of ADC and FF measurements in focal bone marrow lesions will be highly influential in guiding biomarker development in bone marrow, and the influence of lesion size is unknown.

Our results show for ADC-Mean in both the small and large lesion groups, intra- and inter-reader estimates of *s_w_* are similar with low CoV and excellent ICC. This indicates that for ADC-Mean multiple measurements can be obtained by more than one reader. ADC-SD was found to be greater in larger lesions. As ADC-SD is a measure of the heterogeneity it is possible that greater tissue differentiation in larger compared with smaller lesions would lead to higher values. Inter reader variability is higher than intrareader variability for both size groups. This suggests that a single reader is better able to consistently measure the heterogeneity of ADC values. ADC-SD will be more sensitive than ADC-Mean to the exact location of the ROI boundary because small changes in the ROI contour will include/exclude background voxels (which must have a different ADC to the lesion if the lesion can be localized), and the standard deviation statistic is more sensitive to values in the tails of the ROI distribution than the mean.

Our data show that there are no significant differences in the observer agreement of ADC estimates for large and small lesions down to the 5 mm threshold evaluated in this study.

The Metastases guidelines for WB-MRI in advanced prostate cancer (MET-RAD-P) recommend a 15 mm threshold for lesion size measurement to account for the spatial resolution of MRI but at the spatial resolution used in this study (3D, fast spin-echo sequences) the 15 mm threshold can be reduced to 5 mm.^8^ However, due to lack of available data to date, there are no specific recommendations for ADC measurement in terms of ROI and lesion size in current WB-MRI guidelines.^8,9^

However, with regards to FF-Mean our data suggest that quantitative estimates are more error-prone when obtained from smaller lesions. The findings that: (i) FF-Mean s_W_ estimates are higher for small lesions than large lesions, (ii) ROIs for small lesions drawn on FF maps are smaller than those drawn on ADC maps; are consistent with the hypothesis that ROIs drawn on FF maps tend to exclude peripheral tumour tissue because it has a similar FF to the surrounding tissues. This interpretation assumes that there are no plausible biological reasons for small lesions to have higher FFs.

Taken together these results suggest that observer agreement is significantly impaired when measuring FF of lesions less than 10 mm in diameter. This may be caused by differences in FF between the periphery and interior of the tumour that are difficult to differentiate from the background, leading to upward bias of FF-Mean for small tumours when the additional material is not included, and additional sensitivity to the exact contour chosen leading to increased variability of repeated measures.

This is the first study to assess measurement reproducibility of focal lesions as opposed to background marrow in myeloma. A previous study assessed FF and ADC measurement repeatability in 10 healthy volunteers, scanned up to 4 weeks apart, using seven single slice skeletal 3 cm^2^ ROIs on coronal images (T10, L4, sacral ala, iliac crest, femoral head and neck, mid femur, distal femur) and reported excellent FF repeatability (ICC 0.98), better than ADC (ICC 0.47).^24^ They did not assess reproducibility. However fixed circular ROI measurement in “normal” bone would be easier to perform than manual contouring of focal lesions. Interestingly our results showed excellent ADC-Mean and FF-Mean observer repeatability and reproducibility for both small and large lesions (ICC 0.975–0.982 and ICC 0.937–0.963 respectively).

It should be noted that the T1 weighting from the higher flip angle used for Dixon introduces a bias on FF estimates. However, this approach allows FF measurements to be acquired by making use of the *T*_1_W Dixon images that are already being acquired for anatomical imaging within the MY-RADS guidelines. The addition of proton density Dixon for FF estimates would result in a prohibitive acquisition time for WB-MRI. As such we are assessing FF repeatability and reproducibility in this particular context. A potential limitation of our study is that single slice ROI measurement was used as opposed to whole tumour ROI/volume of interest. Whole tumour ROI ADC measurement has been suggested in rectal cancer to be more reproducible than single slice method; however, they observed no significant difference between tumour ADC and SD between whole tumour and single slice approach.^14^ Myeloma lesions tend to be smaller but more numerous than rectal tumours, so since a single slice approach is less time consuming, this may facilitate clinical adoption. However, were segmentation tools to progress in future such that volumetric whole tumour rather than single slice methods became more commonplace, this could be assessed in future studies. Another limitation is the use of two observers, rather than a larger number, which would give a broader view on the segmentation variability. The finding that inter-reader agreement is only slightly worse in most cases that the intra-reader agreement suggests that the dominant source of variability is the variation between patients, but it would be informative to assess this directly with a study involving more than two readers. In addition, the timing and order in which ROIs were drawn was not pre-specified or documented.

In conclusion, there is excellent intra- and inter-reader agreement for ADC-Mean for both large and small lesions with no evidence of a size effect. For FF measurement, differences in group means coupled with deterioration of intra- and inter-reader agreement in smaller lesions, suggests lesions > 10 mm should be selected for lesion fat fraction measurement. These findings will be helpful in the development of WB-MRI for quantitative response assessment in bone disease.
